# Microbiological Quality of Roasted Coffee and Survival Dynamics of Dry‐Inoculated Bacteria During Storage

**DOI:** 10.1111/1750-3841.71483

**Published:** 2026-09-16

**Authors:** Dionisio Pedro Amorim‐Neto, Jaqueline Sousa Correia, Matheus Péricles Silva Láscaris, Carla Alves Barbosa, Bárbara Cipulo Legabão, Angelica Zaninelli Schreiber, Anderson S. Sant'Ana

**Affiliations:** ^1^ Department of Food Science and Nutrition Faculty of Food Engineering University of Campinas Campinas São Paulo Brazil; ^2^ Department of Pathology Faculty of Medical Sciences University of Campinas Campinas São Paulo Brazil

**Keywords:** challenge test, dry inoculation, desiccation, low‐moisture foods, low water activity, microbial quality

## Abstract

This study evaluated the microbiological quality of 78 roasted coffee samples commercialized in Brazil, including whole‐bean and ground products, and investigated the survival of bacterial species of food safety importance in roasted ground coffee during storage. Although all samples complied with Brazilian legislation, roasted coffee exhibited a diverse microbiological profile, including spoilage microorganisms, coliforms, mesophilic bacteria, and aerobic mesophilic sporeformers. In contrast, *Salmonella*, *Escherichia coli*, *Staphylococcus aureus*, *Bacillus cereus* spores, and thermophilic sporeformers were not detected. MALDI‐TOF analysis identified a predominance of *Bacillus* and *Staphylococcus* species, including opportunistic microorganisms such as *Klebsiella pneumoniae* and *Staphylococcus epidermidis*. A dry inoculation challenge test demonstrated that both species remained viable in vacuum‐packaged roasted ground coffee stored at 25°C for up to 42 days, exceeding the recommended post‐opening shelf life. Weibull modeling indicated good fit for the survival curves and revealed distinct survival behaviors between microorganisms. Despite the persistence observed under low water activity conditions, coffee brewing with hot water resulted in non‐detectable levels of both microorganisms throughout storage.

**Practical Applications:**

This study showed that roasted coffee, whether whole bean or ground, can carry different types of microorganisms, including some capable of forming spores. Certain bacteria (*S. epidermidis* and *K. pneumoniae*) were able to remain alive in roasted coffee for more than 42 days at 25°C, demonstrating their ability to survive in this low‐moisture environment. However, neither microorganism was detected in the final beverage after coffee brewing. These findings reinforce the importance of good hygiene and handling practices during coffee production and commercialization to maintain hygienic quality and minimize potential concerns for vulnerable populations.

## 1 Introduction

Coffee is a globally important beverage (Kim et al. 2026) derived from the fruit of the coffee plant (genus *Coffea*), whose processed seeds are used for beverage production, and Brazil is the leading producer, accounting for 35% of global coffee production (FAS/USDA 2026). Its production is mainly divided between Arabica and Robusta coffee, and in 2025 Brazil produced approximately 56.5 million 60‐kg bags of coffee (ABIC 2026).

In a broader perspective, ripe coffee fruits, known as coffee cherries, are harvested and processed, and the obtained seeds undergo roasting before being commercialized as whole beans or ground coffee (Ferreira et al. 2023; Gomes et al. 2024).

Previous research has largely focused on the microbial diversity of coffee (Veloso et al. 2020; Shen et al. 2024) and the effects of microbial metabolites on coffee quality (da Silva Vale et al. 2022; Shen et al. 2023), whereas studies addressing foodborne pathogens are still limited (Lu et al. 2022). On the other hand, previous studies have shown that coffee production environments can harbor a variety of microorganisms that, besides contaminating the products, may also pose risks to workers due to the formation of contaminated dust and aerosols (Abaya et al. 2018, 2020; Viegas et al. 2022).

However, previous studies have demonstrated that different bacterial species of food and clinical importance are able to contaminate other low water activity foods like coffee, such as cocoa (Iacumin et al. 2022) and guarana powder (Lima et al. 2012; Amorim‐Neto et al. 2025), which are also fermented and roasted products. This reinforces the need to further investigate the microbiological profile of roasted coffee. And to the authors’ knowledge, no previous challenge test has specifically investigated the survival of potential pathogenic microorganisms in roasted coffee during storage and subsequent beverage preparation.

Therefore, the aim of this study was to evaluate the microbiological quality of roasted ground and whole‐bean coffee commercialized in Campinas, São Paulo, Brazil. In addition, the persistence of pathogenic and spoilage microorganisms isolated from the samples was evaluated through their dry re‐inoculation into roasted ground coffee samples.

## 2 Materials and Methods

### 2.1 Roasted Coffee Samples

A total of 78 roasted coffee samples were obtained from retail outlets in Campinas, São Paulo, Brazil. The sampling comprised 26 distinct coffee products from 20 major commercial brands distributed nationwide. For each product, three independent production batches were sampled, resulting in 78 samples (26 products × 3 batches). An independent batch was defined as the same commercial product obtained from a distinct production lot. Some brands contributed more than one of the 26 products because both ground and whole‐bean roasted coffee were sampled when available. Among the products available on the market, six brands were purchased in bulk as whole‐bean and ground coffee (without vacuum packaging), whereas the remaining 14 brands were purchased as vacuum‐packaged ground coffee.

### 2.2 Microbiological Quality of Roasted Coffees

For microbiological analyses, 25 g of roasted coffee samples were diluted in 225 mL of 0.1% (w/v) peptone (Kasvi, Brazil) water or buffered peptone water (BPW, Kasvi, Brazil), depending on the specific requirements of each method, unless other observations are made. Mold and yeast counts were determined by spreading 1 mL onto three plates (300, 300, and 400 µL aliquots) of Dichloran Glycerol 18 (DG18; Neogen, United States), followed by incubation at 25°C for 5 days (Ryu and Wolf‐Hall 2015). Enumeration of *Staphylococcus aureus* was performed by plating the same volumes onto Baird‐Parker agar (BP; Merck, Germany) supplemented with egg yolk and tellurite (Bennett et al. 2015) and incubating at 35°C for 48 h. When detected, black colonies were further identified using matrix‐assisted laser desorption/ionization time‐of‐flight mass spectrometry (MALDI‐TOF MS). Total coliforms and *Escherichia coli* were enumerated using Petrifilm 6404 plates (AOAC 991.14; 3 M, Minnesota, USA), inoculated with 1 mL of sample, and incubated at 35°C for 24–48 h (Kornacki et al. 2015). Aerobic mesophilic bacteria were enumerated by spreading 1 mL onto three plates (300, 300, and 400 µL aliquots) of Plate Count Agar (PCA; KASVI, Brazil) (Ryser and Schuman 2015), followed by incubation at 35°C for 48 h, after which all colonies were counted. Mesophilic spore‐forming bacteria were enumerated from the first decimal dilution following heat treatment at 80°C for 10 min (Stevenson and Lembke 2015). The same heat‐treated dilution was also used for the enumeration of *Bacillus cereus* spores by spread plating 1 mL onto three plates (300, 300, and 400 µL aliquots) of Mannitol Egg Yolk Polymyxin (MYP; Neogen Corporation, Lansing, MI, USA; polymyxin, Sigma‐Aldrich, San Luis, MO, USA) agar, followed by incubation at 30°C for 24 h (ISO 2004); if detected, presumptive colonies were confirmed by MALDI‐TOF MS. Aerobic mesophilic spore‐forming bacteria were further quantified by adding 10 mL of the same heat‐treated dilution to 100 mL of molten Tryptone Glucose Extract agar (TGE; Kasvi, Brazil), which was then poured into five Petri dishes for each sample and incubated at 35°C for 48 h (Stevenson and Lembke 2015). For the enumeration of aerobic thermophilic spore‐forming bacteria, 20 g of sample were homogenized with 100 mL of sterile water, and a 10 mL aliquot was then transferred to 100 mL of molten Dextrose Tryptone Agar (DTA; Merck, Germany) and subjected to boiling for 30 min (Olson and Sorrells 2015). After cooling, the medium was dispensed into five Petri dishes and incubated at 55°C for 72 h. For *Salmonella* detection, samples (25 g) were first pre‐enriched in 225 mL of BPW and incubated at 37°C for 20 h. Aliquots were then subjected to selective enrichment in RVS broth (0.1 mL; Merck, Germany) at 41.5°C for 24 h and in MKTTn broth (1 mL; Oxoid Limited, United Kingdom) at 37°C for 24 h. Cultures from both enrichments were streaked onto Xylose Lysine Deoxycholate (XLD; Neogen, United States) and Brilliant Green agar (Kasvi, Brazil), followed by incubation at 37°C for 24 h (ISO 2017). If present, presumptive colonies were isolated and identified by MALDI‐TOF MS. The detection limit for mesophilic and thermophilic spore‐forming bacterial groups was 1 spore/g. For total coliforms, *E. coli*, *S. aureus*, *B. cereus* spores, aerobic mesophilic bacteria, and molds and yeasts, the detection limit was 10 CFU/g. *Salmonella* was assessed qualitatively and classified as present or absent in 25 g of sample.

### 2.3 Identification of Isolates by MALDI‐TOF

Representative or presumptive colonies of the investigated pathogens were identified by MALDI‐TOF MS. For this purpose, bacterial colonies were directly transferred onto the wells of the Microflex LT steel target plate (Bruker Daltonics, Germany). Subsequently, 1 µL of 70% formic acid was added to each sample and allowed to dry at room temperature. After drying, 1 µL of α‐cyano‐4‐hydroxycinnamic acid (CHCA) matrix solution was applied and again allowed to dry. During analysis, laser ionization/desorption generated protein spectra based on mass‐to‐charge (m/z) ratios. Spectra were acquired using FlexControl v3.4 software (Bruker Daltonics) and compared with the instrument's internal database, enabling bacterial identification according to genus‐ and species‐level confidence scores (Giordano et al. 2023). According to the manufacturer's criteria, MALDI‐TOF MS scores of 2.300–3.000 indicated highly probable species identification, whereas scores of 2.000–2.299 indicated secure genus and probable species identification.

### 2.4 Challenge Test for Microbial Survival in Roasted Ground Coffee

#### 2.4.1 Dry Inoculation and Storage Conditions

*Staphylococcus epidermidis* (LMQA‐RoaCoff‐02) and *Klebsiella pneumoniae* (LMQA‐RoaCoff‐01) isolates were streaked onto Brain Heart Infusion agar (BHI; Himedia Laboratories, Mumbai, India) and incubated at 37°C for 20 h. Colonies were then re‐streaked to obtain confluent surface growth and incubated under the same conditions to maximize active cell recovery.

Dry inoculation was performed using homologous carriers (Amorim‐Neto and Nascimento 2026), adapting the method of Li et al. (2024). Briefly, cells were collected from agar plates using 0.85% (w/v) NaCl (1 mL per plate) and combined to obtain the slurry. Each 1 mL aliquot was used to inoculate 10 g of roasted ground coffee (carrier contamination levels of 8.3 ± 0.08 and 9.4 ± 0.05 log CFU/g for *S. epidermidis* and *K. pneumoniae*, respectively), which was dried in a laminar flow cabinet for approximately 3 h. The contaminated carrier was then mixed with roasted ground coffee (decontaminated for 30 min under UV light in a flow cabinet) at a ratio of 10:90 (carrier:matrix, w/w), reaching an initial contamination level of 7.3 ± 0.20 log CFU/g for S. epidermidis (a_w_ 0.407 ± 0.009) and 8.5 ± 0.20 log CFU/g for *K. pneumoniae* (a_w_ 0.428 ± 0.02). Notably, these high concentrations were used to maximize cell recovery after heat treatment and should not be interpreted as representative of likely consumer exposure. After homogenization, samples were maintained under laminar flow until water activity approached the original product values. The inoculated material was portioned into 30 g portions, vacuum‐sealed in sterile plastic bags, and stored at 25°C for up to 42 days to simulate ambient storage conditions, considering a typical post‐opening shelf life of 30 days. The experiment was conducted in triplicate.

#### 2.4.2 Microbial Enumeration and Coffee Brewing

At each sampling point during storage, one sealed package was removed for each microorganism. Subsequently, 10 g were separated for analysis before heat treatment, and an additional 10 g were subjected to thermal processing. Coffee was prepared by conventional drip brewing using a commercially available single‐use paper filter (GODREAM, 7.5 × 9 cm). The filter was placed over a sterile beaker, and roasted ground coffee (10 g), previously inoculated with the target microorganisms, was brewed with 90 g of boiling water, preheated in an electric kettle (Electrolux). After dripping was complete, the brewed coffee was immediately cooled in an ice bath. The filtrate was collected in a sterile beaker and considered the initial dilution. For microbial enumeration before brewing, 10 g of inoculated coffee were mixed with 90 g of sterile distilled water, serially diluted, and 100 µL of the appropriate dilutions were spread‐plated. For brewed coffee, a total of 1 mL of the resulting filtrate was plated across three agar plates by distributing aliquots of 300, 300, and 400 µL. Baird‐Parker agar was used for *S. epidermidis* and MacConkey agar for *K. pneumoniae* enumerations. The detection limits were 100 CFU/g for inoculated coffee before brewing and 10 CFU/g for brewed filtered coffee. Plates were incubated at 37°C for 24 h.

#### 2.4.3 Model Fitting and Statistical Analysis

Model fitting was performed with the Geeraerd and Van Impe Inactivation Model Fitting Tool (GInaFiT) version 1.7 in Excel version 2470 (Microsoft, Redmond, WA, USA) (Geeraerd et al. 2005). For each microorganism in nontreated roasted coffee, survival curves (Figure 2) were fitted using the Weibull model (Equation 1) (Mafart et al. 2002) as follows:

(1) $$\log\left(\frac{N_{t}}{N_{0}}\right)=-{\left(\frac{t}{\delta }\right)}^{\beta },\;for\;t\;>0$$

Where, δ, is time required to achieve the first decimal reduction (days), β, is the model parameter describing the shape of the curve, t4, is time required for four decimal reductions, R2, is the regression coefficient, and RMSE, mean sum of square error (Table 2).

### 2.5 Physicochemical Analysis

For pH determination, 10 g of each sample were homogenized in 90 mL of distilled water, and measurements were taken using a calibrated pH meter (model K39‐2014B; KASVI, China) (IAL 2005). Water activity (a_w_) was assessed with an AquaLab moisture analyzer (model CX‐2; Decagon Devices, Washington, USA).

## 3 Results and Discussion

**FIGURE 1:**
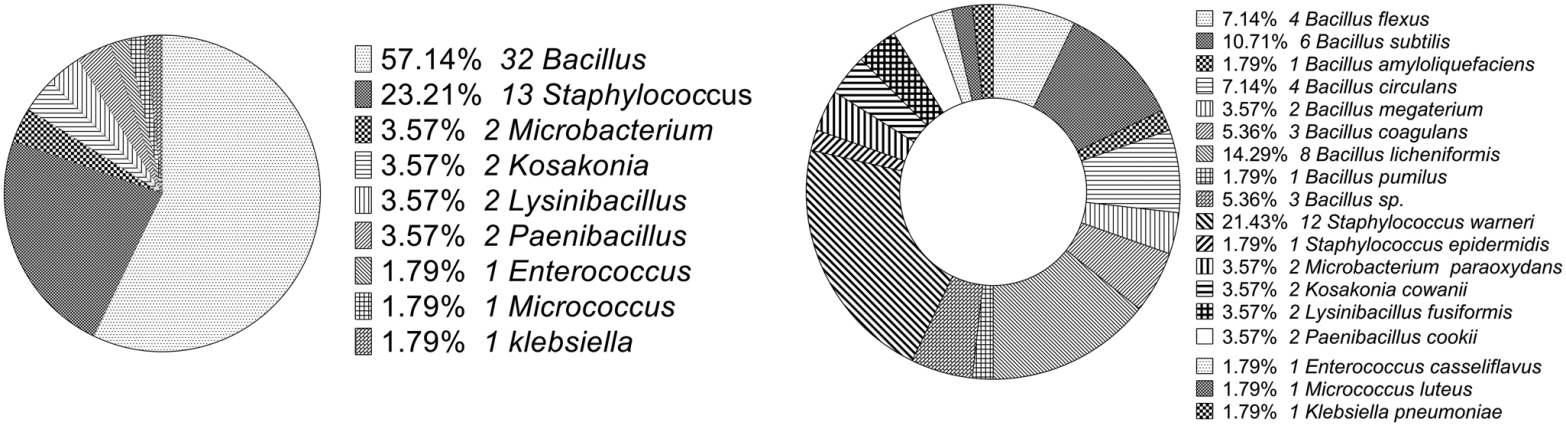
Identification of bacterial isolates (*n =* 56) belonging to microbial groups with microorganisms that are indicators of good manufacturing practices, spoilage organisms, and pathogenic and/or opportunistic potential at the a) genus and b) species levels. Each genus and species is represented by a distinct hatch pattern, with its relative frequency (%) and corresponding number of isolates (n) indicated.

**FIGURE 2:**
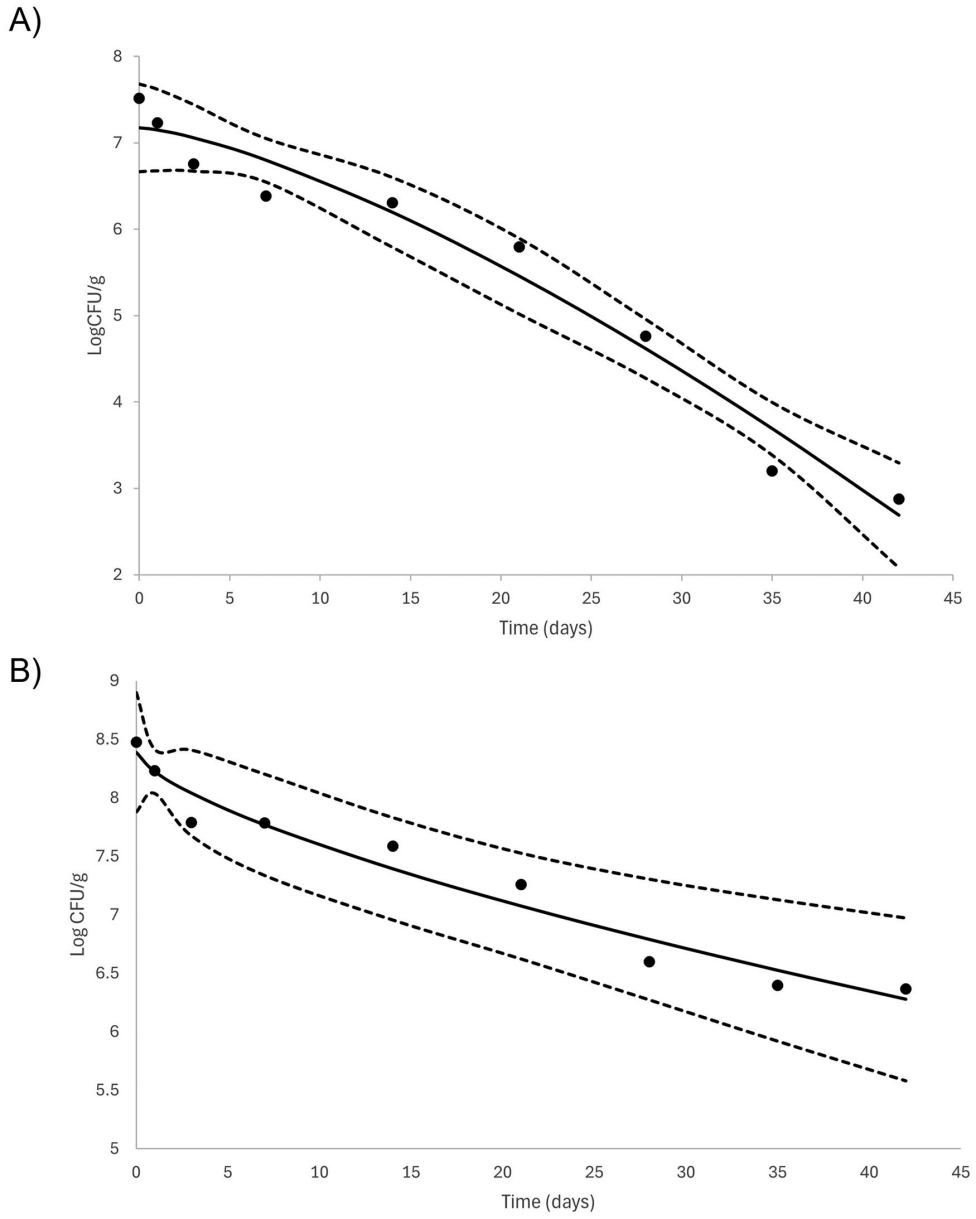
Survival curves of *Staphylococcus epidermidis* (LMQA‐RoaCoff‐02) and *Klebsiella pneumoniae* (LMQA‐RoaCoff‐01) artificially dry‐inoculated into ground roasted coffee, vacuum‐packaged, and stored at 25°C. Results expressed as means with the 95 % confidence intervals.

In this study, the microbiological quality of roasted ground and whole‐bean coffee samples from major brands commercialized in Brazil and available at retail outlets in Campinas, São Paulo, was evaluated. Although microbial counts were relatively low (Table 1), several samples showed the presence of spoilage bacteria, microorganisms of hygienic relevance, including opportunistic species (Figure 1). In addition, a challenge test based on a dry inoculation method demonstrated the ability of two previously isolated species of relevance, *Klebsiella pneumoniae* and *Staphylococcus epidermidis*, to persist in roasted ground coffee matrices beyond the product shelf life when stored at room temperature (25°C) (Table 2 and Figure 2).

**TABLE 1:** Microbiological quality* and physicochemical parameters+ of commercial roasted coffee (*n =* 78 samples)^#^.

S	B	Physical form	Vacuum packed	Molds and yeasts	Mesophilic bacteria	Coliforms	*E. coli*	*S. aureus*	*B. cereus* spores	Aerobic Mesophilic spore‐forming bacteria	Aerobic Thermophilic spore‐forming bacteria	a_w_	pH
A	1	Beans	No	<DL	0.9 ± 0.8	<DL	<DL	<DL	<DL	0.5 ± 0.2	<DL	0.614 ± 0.006	5.87 ± 0.09
B		Ground	No	<DL	<DL	<DL	<DL	<DL	<DL	<DL	<DL	0.491 ± 0.020	5.29 ± 0.01
C	2	Beans	No	1.1 ± 0.2	<DL	<DL	<DL	<DL	<DL	0.4 ± 0.0	<DL	0.414 ± 0.022	5.37 ± 0.01
D		Ground	No	<DL	<DL	<DL	<DL	<DL	<DL	<DL	<DL	0.341 ± 0.034	5.09 ± 0.06
E	3	Beans	No	<DL	1.1 ± 0.2	<DL	<DL	<DL	<DL	<DL	<DL	0.483 ± 0.003	6.35 ± 0.03
F		Ground	No	<DL	2.2 ± 0.2	1.0 ± 0.0	<DL	<DL	<DL	0.4 ± 0.0	<DL	0.505 ± 0.001	6.42 ± 0.01
G	4	Beans	No	1.0 ± 0.0	1.5 ± 0.2	<DL	<DL	<DL	<DL	0.9 ± 0.3	<DL	0.489 ± 0.003	5.60 ± 0.02
H		Ground	No	<DL	0.3 ± 0.6	<DL	<DL	<DL	<DL	<DL	<DL	0.505 ± 0.002	5.80 ± 0.01
I	5	Beans	No	<DL	<DL	<DL	<DL	<DL	<DL	<DL	<DL	0.481 ± 0.003	5.89 ± 0.01
J		Ground	No	<DL	<DL	<DL	<DL	<DL	<DL	<DL	<DL	0.497 ± 0.001	6.05 ± 0.01
K	6	Beans	No	1.0 ± 0.0	<DL	<DL	<DL	<DL	<DL	<DL	<DL	0.438 ± 0.002	5.36 ± 0.02
L		Ground	No	<DL	1.3 ± 0.3	<DL	<DL	<DL	<DL	<DL	<DL	0.363 ± 0.023	5.38 ± 0.03
M	7	Ground	Yes	<DL	<DL	<DL	<DL	<DL	<DL	<DL	<DL	0.385 ± 0.002	6.94 ± 0.09
N	8	Ground	Yes	<DL	<DL	<DL	<DL	<DL	<DL	<DL	<DL	0.427 ± 0.001	6.10 ± 0.08
O	9	Ground	Yes	<DL	0.7 ± 0.6	<DL	<DL	<DL	<DL	<DL	<DL	0.481 ± 0.002	5.46 ± 0.03
P	10	Ground	Yes	<DL	<DL	<DL	<DL	<DL	<DL	<DL	<DL	0.368 ± 0.002	5.94 ± 0.01
Q	11	Ground	Yes	<DL	<DL	<DL	<DL	<DL	<DL	1.0 ± 0.1	<DL	0.377 ± 0.002	5.78 ± 0.01
R	12	Ground	Yes	<DL	<DL	<DL	<DL	<DL	<DL	<DL	<DL	0.411 ± 0.001	5.69 ± 0.01
S	13	Ground	Yes	<DL	0.8 ± 0.8	<DL	<DL	<DL	<DL	1.9 ± 0.5	<DL	0.424 ± 0.002	5.65 ± 0.01
T	14	Ground	Yes	<DL	1.2 ± 0.3	0.7 ± 0.6	<DL	<DL	<DL	2.1 ± 0.2	<DL	0.406 ± 0.004	5.95 ± 0.02
U	15	Ground	Yes	<DL	<DL	<DL	<DL	<DL	<DL	1.1 ± 0.1	<DL	0.467 ± 0.001	5.62 ± 0.01
V	16	Ground	Yes	<DL	1.2 ± 0.3	<DL	<DL	<DL	<DL	0.4 ± 0.0	<DL	0.355 ± 0.002	5.64 ± 0.01
X	17	Ground	Yes	<DL	<DL	<DL	<DL	<DL	<DL	<DL	<DL	0.444 ± 0.002	5.76 ± 0.01
W	18	Ground	Yes	<DL	<DL	0.7 ± 0.6	<DL	<DL	<DL	<DL	<DL	0.422 ± 0.012	6.04 ± 0.01
Y	19	Ground	Yes	<DL	<DL	<DL	<DL	<DL	<DL	1.0 ± 0.2	<DL	0.459 ± 0.001	5.77 ± 0.01
Z	20	Ground	Yes	<DL	2.2 ± 0.1	<DL	<DL	<DL	<DL	2.4 ± 0.1	<DL	0.420 ± 0.006	6.06 ± 0.02

*Note*: Data are expressed as mean (*n =* 3) ± standard deviation mean of log10 CFU or spores per g/mL of sample.

#Derived from the analysis of three batches, with 26 samples per batch (78 samples total).

+Results are expressed as mean ± standard deviation.

Abbreviations: B, Brands; <DL, below the detection limit; S, Samples.

**TABLE 2:** Parameters of the Weibull model fitted for *Staphylococcus epidermidis* (LMQA‐RoaCoff‐02) and *Klebsiella pneumoniae* (LMQA‐RoaCoff‐01) survival in ground roasted coffee during a 42 days storage period at 25°C.

	δ (days)	β	t_4_ (days)	R^2^	RMSE
*Staphylococcus epidermidis*	14 ± 1.68	1.39	36.62 ± 0.11	0.96	0.37
*Klebsiella pneumoniae*	14.1 ± 3.44	0.68	124.12 ± 44.10	0.96	0.18

*Note*: δ, time required to achieve the first decimal reduction (days).

β, model parameter describing the shape of the curve.

t4, time required for four decimal reductions.

R2, regression coefficient.

Data are expressed as the mean of the parameter estimate ± standard deviation (*n =* 3).

Abbreviations: RMSE, mean sum of square error.

Initially, *B. cereus* spores, thermophilic aerobic spore‐forming bacteria, *E. coli*, *S. aureus*, and *Salmonella* were not detected. With respect to Brazilian legislation, all samples complied with regulatory requirements, as the legislation establishes the absence of *Salmonella* and *E. coli* in this type of product (ANVISA 2019). This scenario is similar to that reported by Anosike and Oranusi (2018), who also did not detect *S. aureus* in roasted ground coffee samples purchased from supermarkets in Nigeria, but differs from Chaves et al. (2012), who reported *B. cereus* spores in 56.7% of roasted ground coffee samples commercialized in Brazil (without geographic specification), at levels ranging from 1 to 3 log spores/g.

Three whole‐bean coffee brands showed 1‐1.1 log CFU/g of molds and yeasts, values lower than those reported by Viegas et al. (2022) for coffee bean samples collected directly from workplaces in Minas Gerais State, Brazil, from two different companies (1.41 × 10^2^ CFU/g and 5.34 × 10^2^ CFU/g, respectively).

Aerobic mesophilic bacteria were detected in 3 whole‐bean samples (0.9–1.9 log CFU/g) and 8 ground samples (0.3–2.2 log CFU/g). As a broad microbial group, aerobic mesophilic bacteria have been reported in most of the few available studies investigating the microbiological quality of roasted coffee. For whole‐bean coffee, counts of 2.20 × 10^4^ CFU/g and 1.9 × 10^5^ CFU/g were reported by Viegas et al. (2022) and Abaya et al. (2020), respectively, whereas for ground coffee, counts up to 4.5 × 10^1^ CFU/g were described by Anosike et al. (2018).

Total coliforms were detected in three ground coffee samples (0.7–1 log CFU/g), which may be indicative of hygiene conditions during handling or processing. In addition, among the identified microorganisms belonging to the *Enterobacteriaceae* family, *K. pneumoniae* and *Kosakonia cowanii* were detected (Figure 1), with *K. pneumoniae* being an opportunistic pathogen within the coliform group. In this context, Viegas et al. (2022) also reported Enterobacteriaceae counts of 2.52 × 10^3^ CFU/g in roasted coffee bean samples.

Finally, mesophilic aerobic spore‐forming bacteria were detected in 4 whole‐bean samples (0.4–0.9 log spores/g) and 7 ground samples (0.4–2.4 log spores/g), representing the highest incidence and contamination levels among the microbial groups analyzed. This finding is consistent with the physicochemical characteristics of the samples (Table 1), which roasted bean coffees presented pH values of 5.74 ± 0.38 and water activity of 0.487 ± 0.063, while for roasted ground coffee, it was 5.82 ± 0.41 for pH and 0.427 ± 0.052 for water activity, conditions that hinder the maintenance of vegetative cells in the product.

Seasonal variation, geographical origin, and post‐harvest processing are major determinants of the coffee microbiota and, consequently, of the microbiological characteristics of the final product. Environmental factors such as altitude (Veloso et al. 2020), temperature (Góngora et al. 2024), and edaphoclimatic conditions (Simmer et al. 2022) shape bacterial and fungal communities from cultivation through fermentation, resulting in region‐specific microbial assemblages and dominant taxa across coffee‐producing countries (Veloso et al. 2020; Góngora et al. 2024; Shen et al. 2025; de Melo Pereira et al. 2026). Post‐harvest practices further influence this microbial diversity, as wet and dry processing (Hamdouche et al. 2016), washed (Shen et al. 2023), semi‐dry (Shen et al. 2024), and dry (Shen et al. 2025) methods, together with anaerobic and extended fermentations, select distinct microbial communities and metabolite profiles (da Silva Vale et al. 2022; Silva et al. 2024). Seasonality also contributes to these differences, with harvest period, fruit maturity, and drying conditions affecting microbial contamination and community composition, while regional characteristics further modulate these responses (Simmer et al. 2022; Holguín‐Sterling et al. 2023; Tenea et al. 2025; Abreu et al. 2025). Future studies should include region‐specific coffee products, considering the diversity of Brazilian coffee‐producing regions and their processing, storage, and commercialization practices, as well as a broader range of foodborne pathogens to improve the representativeness and applicability of the findings.

Indeed, the selective pressure imposed by the low water activity of coffee samples was reflected in the bacterial contamination profile (Figure 1). A total of nine bacterial genera were identified, with a predominance of *Bacillus* and *Staphylococcus*. At the species level, among the 18 species identified, *Staphylococcus warneri* (21.43%), *Bacillus licheniformis* (14.29%), and *Bacillus subtilis* (10.71%) were the most prevalent. Notably, *Bacillus* subtilis spores were also widely reported by Chaves et al. (2012) in roasted ground coffee samples.

From a broader perspective, many of the species identified in this study (Figure 1) have also been previously reported as contaminants in other fermented foods subjected to thermal processing, such as cocoa and guarana powder (Lima et al. 2012; Iacumin et al. 2022; Amorim‐Neto et al. 2025).

*Staphylococcus epidermidis* and *Klebsiella pneumoniae* were selected for the challenge test because, although neither is considered a classical foodborne pathogen, available evidence provides a rationale for investigating their persistence and survival during food storage and preparation. These species have also been reported in food production environments, with some isolates harboring genes associated with virulence or antimicrobial resistance (Ajis et al. 2017; Chajęcka‐Wierzchowska et al. 2019; Byczkowska‐Rostkowska et al. 2025; Thacharodi et al. 2026). Future studies should characterize the isolates recovered in the present study to determine whether they harbor these traits. Specifically for *S. epidermidis*, preclinical studies in murine models have reported pathological alterations following repeated oral administration of the microorganism or its enterotoxins, including effects on the gastrointestinal tract (Akinkunmi et al. 2014; Tabiś et al. 2022). For *K. pneumoniae*, evidence suggests that food may represent a potential vehicle for its dissemination. *K. pneumoniae* and other members of the *K. pneumoniae* species complex have been detected in chicken meat, salads, and other retail foods, with some isolates carrying virulence determinants and antimicrobial resistance (Zhang et al. 2018; Rodrigues et al. 2022). Meat‐derived isolates have also shown genetic relatedness and similar virulence to clinical isolates, suggesting possible transmission between food and human sources (Davis et al. 2015). In addition, a potential role of food in transmission was reported during a hospital outbreak in which the same *K. pneumoniae* strain was identified among patients, food handlers, and samples from the hospital kitchen (Calbo et al. 2011). Therefore, to further evaluate microbial persistence in roasted ground coffee, isolates of *K. pneumoniae* (LMQA‐RoaCoff‐01) and *S. epidermidis* (LMQA‐RoaCoff‐02) were re‐inoculated into the matrix, vacuum‐sealed, and stored at 25°C for 42 days, remaining viable throughout the entire storage period (Figure 2).

The Weibull model was used to describe bacterial survival in roasted coffee samples, and the high R^2^ values together with the low RMSE values (Table 2) indicated a good fit of the model (Mafart et al. 2002; Geeraerd et al. 2005) to the challenge test data obtained for both microorganisms. Particular attention should be given to the δ values, which represent the time required to achieve the first decimal reduction. Similar δ values were observed for both bacteria, at approximately 15 days, corresponding to nearly half of the product shelf life after opening according to the manufacturer's recommendations. However, the survival behavior during storage differed markedly between the microorganisms (Figure 2). *S. epidermidis* (LMQA‐RoaCoff‐02) exhibited a shoulder‐type inactivation curve (β > 1), whereas *K. pneumoniae* (LMQA‐RoaCoff‐01) showed a tailing‐type profile (β < 1), which corresponds to a faster inactivation rate or greater resistance, respectively (Geeraerd et al. 2005). In addition, the time required to achieve a four‐log reduction in the dry inoculated population was approximately three times greater for *K. pneumoniae* (LMQA‐RoaCoff‐01) than for *S. epidermidis* (LMQA‐RoaCoff‐02), although this parameter represents a model‐based extrapolation beyond the experimental storage period. To the best of the authors’ knowledge, there are no previous studies investigating the behavior of these microorganisms during storage in low‐moisture foods. Nevertheless, the desiccation tolerance observed here is consistent with reports involving other low‐water activity foods, such as guarana powder, dried mushrooms, pistachios, and different flours, in which these species have also been identified in contaminated samples (Al‐Moghazy et al. 2014; Ajis et al. 2017; Koreneková et al. 2022; Amorim‐Neto et al. 2025).

To evaluate whether desiccation stress induced resistance in the inoculated isolates during the product storage, dry contaminated coffee samples were used to prepare coffee beverages by filtration with boiling water. However, both microorganisms were not recovered at any point during the storage period after beverage preparation, indicating that the process was effective in eliminating vegetative cells, with no evidence of resistance.

Although this study did not investigate contamination sources along the production chain, previous HACCP studies (Kourtis and Arvanitoyannis 2001; Lopez‐Garcia et al. 2008; Winkler 2014) have consistently identified post‐roasting handling, grinding, and packaging as critical stages that should be carefully evaluated during hazard analysis because they represent potential sources of post‐process contamination. Accordingly, the coffee industry should integrate routine microbiological monitoring with good manufacturing practices and HACCP‐based hazard analysis throughout post‐roasting operations, including handling, grinding, packaging, storage, and commercialization, to minimize contamination and maintain microbiological quality.

In summary, this study was limited to nationally distributed coffee brands purchased in a single city and evaluated a restricted number of microorganisms in the survival assay. Future studies should also investigate the potential survival of microorganisms in coffee grounds retained by paper filters, waste, and during coffee preparation using alternative brewing methods that do not employ paper filters (e.g., French press, espresso, and Turkish coffee). Because commercially packaged coffee, rather than freshly processed coffee obtained directly from the manufacturer, was used in this study, the manufacturer's recommended post‐opening storage period was adopted. However, this represents a limitation of the experimental design, as the storage conditions did not fully reproduce those encountered after package opening, including exposure to oxygen, humidity fluctuations, repeated handling, and environmental contamination.

## 4 Conclusion

Finally, it was concluded that although roasted ground and whole‐bean coffee samples presented different levels of contamination, all samples complied with Brazilian legislation (ANVISA 2019), which specifically establishes standards for *Salmonella* and *E. coli*, neither of which were detected. Despite the low water activity, roasted coffee exhibited a diverse microbiological contamination profile, including spoilage and opportunistic bacteria detected as vegetative cells or spores. In this regard, the detection of opportunistic microorganisms is relevant from a hygiene perspective, particularly considering vulnerable populations such as immunocompromised individuals, and underscores the importance of good handling practices, especially at points of sale. In addition, dry re‐inoculation of *K. pneumoniae* and *S. epidermidis* isolates into roasted ground coffee stored at room temperature demonstrated that these microorganisms remained viable for periods exceeding the recommended shelf life after package opening. However, neither microorganism was recovered from the brewed coffee filtrate under the tested conditions, with microbial counts remaining below the detection limit.

## Author Contributions

**Dionisio Pedro Amorim‐Neto**: conceptualization, investigation, writing – original draft, writing – review and editing, visualization, validation, methodology, formal analysis, data curation. **Jaqueline Sousa Correia**: investigation, conceptualization, writing – original draft, methodology, validation, visualization, writing – review and editing. **Matheus Péricles Silva Láscaris**: conceptualization, investigation, writing – original draft, visualization, validation, methodology, writing – review and editing. **Carla Alves Barbosa**: conceptualization, investigation, writing – original draft, visualization, validation, methodology, writing – review and editing. **Bárbara Cipulo Legabão**: investigation, writing – original draft, methodology, validation, visualization, writing – review and editing. **Angelica Zaninelli Schreiber**: investigation, writing – original draft, funding acquisition, methodology, validation, writing – review and editing, project administration, software, supervision, resources. **Anderson S. Sant'Ana**: conceptualization, investigation, funding acquisition, writing – original draft, writing – review and editing, visualization, validation, methodology, formal analysis, project administration, supervision, resources.

## Conflicts of Interest

The authors declare no conflicts of interest.

## Data Availability

Data will be made available upon reasonable request.

## References

[jfds71483-bib-0001] Abaya, S. W. , M. Bråtveit , W. Deressa , et al. 2018. “Reduced Lung Function Among Workers in Primary Coffee Processing Factories in Ethiopia: A Cross Sectional Study.” International Journal of Environmental Research and Public Health 15: 2415. 10.3390/ijerph15112415. 30384429 PMC6266034

[jfds71483-bib-0002] Abaya, S. W. , M. Bråtveit , W. Deressa , et al. 2020. “Microbial Contamination of Coffee During Postharvest on Farm Processing: A Concern for the respiratory Health of Production Workers.” Archives of Environmental & Occupational Health 75: 201–208. 10.1080/19338244.2019.1592094. 30929620

[jfds71483-bib-0003] ABIC, Associação Brasileira da Indústria de Café . 2026. “Histórico da produção agrícola de café no Brasil.” Accessed 8, May. https://www.abic.com.br/estatisticas/producao‐agricola/.

[jfds71483-bib-0004] Abreu, D. J. M. D. , M. S. Lorenço , and G. G. L. Machado , et al. 2025. “Influence of Drying Methods on the Post‐Harvest Quality of Coffee: Effects on Physicochemical, Sensory, and Microbiological Composition.” Foods 14: 1463. 10.3390/foods14091463. 40361545 PMC12071231

[jfds71483-bib-0005] Ajis, A. H. , L. J. Chong , Y. S. Tan , and L. C. Chai . 2017. “Microbial Food Safety Assessment of Dried Edible Mushrooms.” Journal of Consumer Protection and Food Safety 12: 265–269. 10.1007/s00003-017-1117-x.

[jfds71483-bib-0006] Akinkunmi, E. O. , O. I. Adeyemi , O. A. Igbeneghu , et al. 2014. “The Pathogenicity of *Staphylococcus epidermidis* on the Intestinal Organs of Rats and Mice: An Experimental Investigation.” BMC Gastroenterology [Electronic Resource] 14: 126. 10.1186/1471-230X-14-126. 25016472 PMC4105098

[jfds71483-bib-0007] Al‐Moghazy, M. , S. Boveri , and A. Pulvirenti . 2014. “Microbiological Safety in Pistachios and Pistachio Containing Products.” Food Control 36: 88–93. 10.1016/J.FOODCONT.2013.07.030.

[jfds71483-bib-0008] Amorim‐Neto, D. P. , C. M. G. Lima , S. Ramos , N. M. de , et al. 2025. “The Culturable Microbiological Landscape of Guarana‐Based Products and Wastes: Insights From the Amazon state (Brazil) Production Chain.” Applied Food Research 5: 101162. 10.1016/j.afres.2025.101162.

[jfds71483-bib-0009] Amorim‐Neto, D. P. , and M. S. Nascimento . 2026. “Dry Inoculation Models for *Salmonella* in Low‐Moisture Foods: Challenges, Stress Adaptation, and Biofilm Formation.” Current Opinion in Food Science 68: 101410. 10.1016/J.COFS.2026.101410.

[jfds71483-bib-0010] Anosike, S. O. , and S. Oranusi . 2018. “Assessment of microbiological and chemical qualities of selected cocoa, tea and coffee brands in Nigerian markets.” African Journal of Clinical & Experimental Microbiology 19, no. 3: 186. 10.4314/ajcem.v19i3.5.

[jfds71483-bib-0011] ANVISA, Agência Nacional de Vigilância Sanitária . 2019. “Instrução Normativa N° 60, de 23 de Dezembro de 2019.” In Diário Oficial da União, Edição 249, Seção 1. Imprensa Nacional. https://www.in.gov.br/en/web/dou/‐/instrucao‐normativa‐n‐60‐de‐23‐de‐dezembro‐de‐2019‐235332356.

[jfds71483-bib-0012] Bennett, R. W. , J. M. Hait , and S. M. Tallent . 2015. “ *Staphylococcus* Aureus and Staphylococcal Enterotoxins.” In Compendium of Methods for the Microbiological Examination of Foods, edited by Y. Salfinger , and T. M. Lou , 509–526. American Public Health Association.

[jfds71483-bib-0013] Byczkowska‐Rostkowska, Z. , J. Gajewska , and W. Chajęcka‐Wierzchowska . 2025. “Whole Genome Analysis and Antimicrobial Resistance Assessment of *Staphylococcus epidermidis* Isolated From Food Sources.” Science of The Total Environment 993: 179999. 10.1016/j.scitotenv.2025.179999. 40592212

[jfds71483-bib-0014] Calbo, E. , N. Freixas , M. Xercavins , et al. 2011. “Foodborne Nosocomial Outbreak of SHV1 and CTX‐M‐15–Producing *Klebsiella pneumoniae*: Epidemiology and Control.” Clinical Infectious Diseases 52: 743–749. 10.1093/CID/CIQ238. 21367727

[jfds71483-bib-0015] Chajęcka‐Wierzchowska, W. , A. Zadernowska , and J. Gajewska . 2019. “S. *epidermidis* Strains From Artisanal Cheese Made From Unpasteurized Milk in Poland—Genetic Characterization of Antimicrobial Resistance and Virulence Determinants.” International Journal of Food Microbiology 294: 55–59. 10.1016/j.ijfoodmicro.2019.02.004. 30771666

[jfds71483-bib-0016] Chaves, J. Q. , F. de , C. Gomes Cavados , and A. M. Vivoni . 2012. “Molecular and Toxigenic Characterization of *Bacillus* Cereus and *Bacillus* Thuringiensis Strains Isolated From Commercial Ground Roasted Coffee.” Journal of Food Protection 75: 518–522. 10.4315/0362-028X.JFP-11-325. 22410226

[jfds71483-bib-0017] da, S. , A. Vale , G. Balla , L. R. S. Rodrigues , et al. 2022. “Understanding the Effects of Self‐Induced Anaerobic Fermentation on Coffee Beans Quality: Microbiological, Metabolic, and Sensory Studies.” Foods 12: 37. 10.3390/foods12010037. 36613253 PMC9818356

[jfds71483-bib-0018] Davis, G. S. , K. Waits , L. Nordstrom , et al. 2015. “Intermingled *Klebsiella pneumoniae* Populations between Retail Meats and Human Urinary Tract Infections.” Clinical Infectious Diseases 61: 892–899. 10.1093/CID/CIV428. 26206847 PMC4551003

[jfds71483-bib-0019] de Melo Pereira, G. V. , S. da , A. Vale , A. I. Ribeiro‐Barros , et al. 2026. “Integrated Microbial–Metabolomic Analysis Reveals How Fermentation Contributes to the Unique Flavor of African Arabica Coffee.” Food Chemistry: Molecular Sciences 12: 100344. 10.1016/J.FOCHMS.2025.100344. 41625959 PMC12859811

[jfds71483-bib-0020] FAS/USDA . 2026. “Foreign Agricultural Service.” United States Department of Agriculture. Coffee Production 2025/2026. https://www.fas.usda.gov/data/production/0711100.

[jfds71483-bib-0021] Ferreira, L. J. C. , G. M. D. S , O. L. M. de , and L. D. Santos . 2023. “Coffee Fermentation Process: A Review.” Food Research International 169: 112793. 10.1016/J.FOODRES.2023.112793. 37254380

[jfds71483-bib-0022] Geeraerd, A. H. , V. P. Valdramidis , and J. F. Van Impe . 2005. “GInaFiT, a Freeware Tool to Assess Non‐Log‐Linear Microbial Survivor Curves.” International Journal of Food Microbiology 102: 95–105. 10.1016/j.ijfoodmicro.2004.11.038. 15893399

[jfds71483-bib-0023] Giordano, A. , L. Pontes , C. A. G. Beraquet , et al. 2023. “Matrix‐Assisted Laser Desorption/Ionisation‐Time of Flight Mass Spectrometry Azole Susceptibility Assessment in Candida and Aspergillus Species.” Memorias Do Instituto Oswaldo Cruz 118: e220213. 10.1590/0074-02760220213. 36921145 PMC10014031

[jfds71483-bib-0024] Gomes W dos, S. , L. L. Pereira , L. J. M. R. da , et al. 2024. “Exploring the Microbiome of Coffee Plants: Implications for Coffee Quality and Production.” Food Research International 179: 113972. 10.1016/J.FOODRES.2024.113972. 38342526

[jfds71483-bib-0025] Góngora, C. E. , L. Holguín‐Sterling , B. Pedraza‐Claros , et al. 2024. “Metataxonomic Identification of Microorganisms During the Coffee Fermentation Process in Colombian Farms (Cesar Department).” Foods 13: 839. 10.3390/foods13060839. 38540829 PMC10969282

[jfds71483-bib-0026] Hamdouche, Y. , J. C. Meile , D. N. Nganou , et al. 2016. “Discrimination of Post‐Harvest Coffee Processing Methods by Microbial Ecology Analyses.” Food Control 65: 112–120. 10.1016/J.FOODCONT.2016.01.022.

[jfds71483-bib-0027] Holguín‐Sterling, L. , B. Pedraza‐Claros , R. Pérez‐Salinas , et al. 2023. “Physical–Chemical and Metataxonomic Characterization of the Microbial Communities Present During the Fermentation of Three Varieties of Coffee From Colombia and Their Sensory Qualities.” Agriculture 13: 1980. 10.3390/agriculture13101980.

[jfds71483-bib-0028] Iacumin, L. , M. Pellegrini , A. Colautti , et al. 2022. “Microbial Characterization of Retail Cocoa Powders and Chocolate Bars of Five Brands Sold in Italian Supermarkets.” Foods 11: 2753. 10.3390/foods11182753. 36140882 PMC9497492

[jfds71483-bib-0029] IAL, Adolfo Lutz Institute . 2005. Physicochemical Methods for Food Analysis: Analytical Standards of the Adolfo Lutz Institute, 4th ed. ANVISA.

[jfds71483-bib-0030] ISO, International Organization for Standardization . 2004. “ISO 7932:2004. Microbiology of Food and Animal Feeding Stuffs—Horizontal Method for the Enumeration of Presumptive *Bacillus* Cereus—Colony‐Count Technique at 30 Degrees C.” https://www.iso.org/standard/38219.html.

[jfds71483-bib-0031] ISO, International Organization for Standardization . 2017. “ISO 6579‐1: 2017. Microbiology of the Food Chain—Horizontal Method for the Detection, Enumeration and Serotyping of *Salmonella* .” 1th ed. *Part 1: Detection of Salmonella spp*. https://www.iso.org/standard/56712.html. 10.1016/j.ijfoodmicro.2018.03.022 29803313

[jfds71483-bib-0032] Kim, J. , A. Kim , and J.‐K. Shin . 2026. “Customers' mindset About Coffee Value in Comparison: ROBOT versus BARISTA.” Journal of Foodservice Business Research 29: 301–319. 10.1080/15378020.2025.2480430.

[jfds71483-bib-0033] Koreneková, J. , M. Krahulcová , K. Cverenkárová , et al. 2022. “Occurrence of Antibiotic Resistant Bacteria in Flours and Different Plant Powders Used in Cuisine.” Foods 11: 3582. 10.3390/foods11223582. 36429175 PMC9689793

[jfds71483-bib-0034] Kornacki, J. L. , J. B. Gurtler , and B. A. Stawick . 2015. “Enterobacteriaceae, Coliforms, and *Escherichia* Coli as Quality and Safety Indicators.” In Compendium of Methods for the Microbiological Examination of Foods, edited by Y. Salfinger , and T. M. Lou , 103–120. American Public Health Association.

[jfds71483-bib-0035] Kourtis, L. K. , and I. S. Arvanitoyannis . 2001. “Implementation of Hazard Analysis Critical Control Point (HACCP) System to the Non‐Alcoholic Beverage Industry.” Food Reviews International 17: 451–486. 10.1081/FRI-100108533.

[jfds71483-bib-0036] Li, S. , W. Xu , M. Lin , et al. 2024. “Heat Resistance, Virulence, and Gene Expression of Desiccation‐Adapted *Salmonella* Enteritidis during Long‐Term Storage in Low‐Water Activity Foods.” Foodborne Pathogens and Disease 21: 119–126. 10.1089/fpd.2023.0101. 38010814

[jfds71483-bib-0037] Lima, L. J. R. , V. van der Velpen , J. Wolkers‐Rooijackers , et al. 2012. “Microbiota Dynamics and Diversity at Different Stages of Industrial Processing of Cocoa Beans Into Cocoa Powder.” Applied and environmental microbiology 78: 2904–2913. 10.1128/AEM.07691-11. 22327588 PMC3318835

[jfds71483-bib-0038] Lopez‐Garcia, R. , C. A. Mallmann , and M. Pineiro . 2008. “Design and Implementation of an Integrated Management System for Ochratoxin A in the Coffee Production Chain.” Food Additives & Contaminants 25: 231–240. 10.1080/02652030801946546. 18286413

[jfds71483-bib-0039] Lu, L. , S. Tibpromma , S. C. Karunarathna , et al. 2022. “Comprehensive Review of Fungi on Coffee.” Pathogens 11: 411. 10.3390/pathogens11040411. 35456086 PMC9024902

[jfds71483-bib-0040] Mafart, P. , O. Couvert , S. Gaillard , and I. Leguerinel . 2002. “On Calculating Sterility in Thermal Preservation Methods: Application of the Weibull Frequency Distribution Model.” International Journal of Food Microbiology 72: 107–113. 10.1016/S0168-1605(01)00624-9. 11843401

[jfds71483-bib-0041] Olson, K. E. , and K. M. Sorrells . 2015. “Thermophilic Flat Sour Sporeformers.” In Compendium of Methods for the Microbiological Examination of Foods, edited by Y. Salfinger , and T. M. Lou , 329–333. American Public Health Association.

[jfds71483-bib-0042] Rodrigues, C. , K. Hauser , N. Cahill , et al. 2022. “High Prevalence of *Klebsiella pneumoniae* in European Food Products: A Multicentric Study Comparing Culture and Molecular Detection Methods.” Microbiology Spectrum 10: e02376‐21. 10.1128/spectrum.02376-21. 35196810 PMC8865463

[jfds71483-bib-0043] Ryser, E. T. , and J. D. Schuman . 2015. “Mesophilic Aerobic Plate Count.” In Compendium of Methods for the Microbiological Examination of Foods, edited by Y. Salfinger , and T. M. Lou , 299–304. American Public Health Association.

[jfds71483-bib-0044] Ryu, D. , and C. Wolf‐Hall . 2015. “Yeasts and Molds.” In Compendium of Methods for the Microbiological Examination of Foods, edited by Y. Salfinger , and T. M. Lou , 209–216. American Public Health Association.

[jfds71483-bib-0045] Shen, X. , B. Wang , C. Zi , et al. 2023. “Interaction and Metabolic Function of Microbiota During the Washed Processing of Coffea Arabica.” Molecules 28: 6092. 10.3390/molecules28166092. 37630344 PMC10458683

[jfds71483-bib-0046] Shen, X. , Q. Wang , B. Yuan , et al. 2025. “Dynamic Changes of Microbial Communities and Chemical Compounds During the Dry Processing of Coffea Arabica.” Food Science & Nutrition 13: e70178. 10.1002/fsn3.70178. 40291928 PMC12022005

[jfds71483-bib-0047] Shen, X. , W. Yuan , Q. Wang , et al. 2024. “Dynamic Changes in Microbial Communities and Chemical Compounds During the Semi‐Dry Fermentation Processing of Coffea Arabica.” Fermentation 10: 435. 10.3390/fermentation10080435.

[jfds71483-bib-0048] Silva, L. C. F. , P. V. R. Pereira , C. M. A. D. da , et al. 2024. “Enhancing Sensory Quality of Coffee: The Impact of Fermentation Techniques on Coffea Arabica Cv.” Catiguá MG2 Foods 13: 653. 10.3390/foods13050653. 38472766 PMC10931400

[jfds71483-bib-0049] Simmer, M. M. B. , C. de Soares da Silva M , L. L. Pereira , et al. 2022. “Edaphoclimatic Conditions and the Soil and Fruit Microbiota Influence on the Chemical and Sensory Quality of the Coffee Beverage.” European Food Research and Technology 248: 2941–2953. 10.1007/s00217-022-04102-y.

[jfds71483-bib-0050] Stevenson, K. E. , and F. Lembke . 2015. “Mesophilic Aerobic Endospore‐Forming Bacilli.” In Compendium of Methods for the Microbiological Examination of Foods, edited by Y. Salfinger , and M. L. Tortorello , 4th ed. 223–228. American Public Health Association.

[jfds71483-bib-0051] Tabiś, A. , M. Gonet , J. Schubert , et al. 2022. “Analysis of Enterotoxigenic Effect of *Staphylococcus* aureus and *Staphylococcus epidermidis* Enterotoxins C and L on Mice.” Microbiological Research 258: 126979. 10.1016/j.micres.2022.126979. 35158299

[jfds71483-bib-0052] Tenea, G. N. , V. Cifuentes , P. Reyes , and M. Cevallos‐Vallejos . 2025. “Unveiling the Microbial Signatures of Arabica Coffee Cherries: Insights Into Ripeness Specific Diversity, Functional Traits, and Implications for Quality and Safety.” Foods 14: 614. 10.3390/foods14040614. 40002058 PMC11854473

[jfds71483-bib-0053] Thacharodi, A. , T. Ahmed , S. Hassan , et al. 2026. “Carbapenem Resistance and Hypervirulence in *Klebsiella pneumoniae* Are Raising Global Public Health Concerns in the 21st Century.” Iscience 29: 114782. 10.1016/j.isci.2026.114782. 41767277 PMC12936834

[jfds71483-bib-0054] Veloso, T. G. R. , S. M. da , C. S. de , W. S. Cardoso , et al. 2020. “Effects of Environmental Factors on Microbiota of Fruits and Soil of Coffea Arabica in Brazil.” Scientific Reports 10: 14692. 10.1038/s41598-020-71309-y. 32895415 PMC7477199

[jfds71483-bib-0055] Viegas, C. , B. Gomes , F. Oliveira , et al. 2022. “Microbial Contamination in the Coffee Industry: An Occupational Menace Besides a Food Safety Concern?” International Journal of Environmental Research and Public Health 19: 13488. 10.3390/ijerph192013488. 36294069 PMC9602572

[jfds71483-bib-0056] Winkler, A. 2014. “Coffee, Cocoa and Derived Products (e.g. Chocolate).” In Food Safety Management: A Practical Guide for the Food Industry, edited by Y. Motarjemi and H. Lelieveld , 251–282. Academic Press. 10.1016/B978-0-12-381504-0.00010-X.

[jfds71483-bib-0057] Zhang, S. , G. Yang , Q. Ye , et al. 2018. “Phenotypic and Genotypic Characterization of *Klebsiella pneumoniae* Isolated From Retail Foods in China.” Frontiers in Microbiology 9: 291639. 10.3389/fmicb.2018.00289. PMC5839057 29545778

